# Sex Differences in Oxidative Stress Concerning Allergic Diseases

**DOI:** 10.3390/biom15101461

**Published:** 2025-10-16

**Authors:** Mattia Cristallo, Fabiana Furci, Marco Casciaro, Sebastiano Gangemi, Eustachio Nettis

**Affiliations:** 1 Department of Precision and Regenerative Medicine and Ionian Area, Regional Reference Center for Allergic and Immunological Diseases, University of Bari Aldo Moro, 70124 Bari, Italy; cristallomattia@gmail.com (M.C.); ambulatorio.allergologia@uniba.it (E.N.); 2 Provincial Healthcare Unit, Section of Allergy, 89900 Vibo Valentia, Italy; fabianafurci@gmail.com; 3 Unit and School of Allergy and Clinical Immunology, Department of Medical Sciences, University Hospital of Messina, Via Consolare Valeria 1, 98125 Messina, Italy; 4 Operative Unit and School of Allergy and Clinical Immunology, Department of Clinical and Experimental Medicine, University of Messina, Via Consolare Valeria 1, 98125 Messina, Italy; gangemis@unime.it

**Keywords:** gender differences, allergic rhinitis, asthma, urticaria, atopic dermatitis, allergic contact dermatitis, oxidative stress, antioxidant, sex hormones

## Abstract

In recent years, the role of sexual hormones in the pathogenesis and progression of various diseases has progressively being established, which attempts to explain immune dimorphism. Whether physiological or pathological, variations in hormones influence the inflammatory response and adaptive systems to control increased productions of reactive oxygen species, reactive nitrogen species, and free radicals. Primary allergic respiratory and skin diseases were taken into consideration, and possible biomarkers of oxidative stress related to sex differences in the onset and development of atopic diseases were analyzed. Understanding how these variables interact with each other, and evaluating the possible common targets, lays the foundation for the development of tailored therapies with an eye to precision medicine.

## 1. Introduction

### 1.1. Sex Differences in Allergy Epidemiology

Differences between male and female subjects have been observed in various aspects of different diseases, from epidemiology and clinical manifestations to prognosis and survival [1]. Clinical and experimental evidence supports the hypothesis that sex and hypothalamic–pituitary hormones modulate the immune system, and variations in these hormones over a lifespan can lead to alterations in the balance of oxidative stress and changes in the type of immune system response [2].

In particular, some X-coded natural immune players, such as TLR7 and 8 and CXCR3, adaptive immune players, such as CD40 ligand, FoxP3, WAS, and IL2RG, and the expression of estrogen/androgen receptors on immunological cells have an important role in the immune response [3]. Understanding the relationship between these elements could explain the different predispositions and manifestations in the development of immune-related diseases and pave the way toward new treatments tailored to individual patients.

Allergy is a multifactorial disease with a significant impact on society [4]. Allergic diseases are not equally distributed among men and women. In fact, there is a prevalence in males before puberty with a reversal from adolescence [5]. Asthma and allergic rhinitis, atopic dermatitis, food and drug allergy, and anaphylaxis are examples of this prevalence. The only disease that shows the same trend with a persistent male predominance is eosinophilic esophagitis [5]. The higher susceptibility for eczema and atopic diseases was explained by a tendency for indoor activities associated with lower endotoxin exposure [6]. Nevertheless, after puberty, female adolescents are more likely to develop respiratory allergies and asthma. This refers not only to the prevalence but also to the severity of the clinical manifestations; indeed, there is a 3:2 ratio for female to male patients of severe food allergy [7] and almost the same in anaphylaxis [8], numbers that cannot be explained by considering only the higher female self-reporting. In this study, we refer to “sex differences” as biologically determined variations between males and females, such as hormonal profiles and immune responses. Here, the term “sex” is used consistently, as the focus is on biological mechanisms underlying oxidative stress and allergic responses.

As reported above, sex hormones but also genetic and epigenetic variations, social and environmental factors (e.g., occupation, smoking), and sex-related comorbidities (e.g., obesity, gastroesophageal reflux disease-GERD, chronic obstructive pulmonary disease-COPD) are important factors in such differences, especially for asthma [9].

### 1.2. Oxidative Stress and Allergy

Oxidative stress, defined as an imbalance between antioxidants (enzymatic and non-enzymatic) and pro-oxidants (superoxide anion radicals, hydrogen peroxide, hydroxyl radical, peroxynitrite) [10], is the basis of numerous inflammatory [11], autoimmune [12], and neoplastic pathologies [13]. The disruption in redox homeostasis with a chronic or excessive production of reactive oxygen species (ROS) can exacerbate pre-existing health conditions, leading to tissue damage. ROS are the by-products of normal metabolic processes in aerobic organisms [14].

ROS are produced during cell metabolism or exposure to irritant compounds (like smoke, allergens, and pollutants), UV, infections, and chronic stress [15].

Antioxidant systems include enzymes such as superoxide dismutase (SOD), catalase (CAT), glutathione peroxidase (GPx), and non-enzymatic antioxidants like hydrophilic uric acid (UA), vitamin C, reduced glutathione, and vitamins A, D, and E [16]. Magnesium (Mg), a cofactor in more than 300 enzymatic reactions, is also reported to have antioxidant activity and to play a role in the glutathione redox system in asthmatic children [17].

When there is an excess of ROS, proinflammatory enzymes (myeloperoxidase, NADPH oxidase, and inducible nitric oxide synthase) are activated, in combination with nuclear factor kappa B (NF-κB), and are responsible for the inflammatory cascade. As a result, there is an overproduction of ROS and of reactive nitrogen species (RNS) [18]. Hypoxic conditions exacerbate the effects of inflammation-related oxidative stress, leading to overproduction of mitochondrial ROS and irreversible intracellular damage.

The organs that are most sensitive to oxidative damage are the skin and lungs, considering the high oxygen exposition. Therefore, oxidative and nitrosative stress are included in the pathogenesis of respiratory and skin allergies. Food allergies, with the accumulation of advanced glycation end products (AGEs) and other carbonyl compounds, are more related to carbonyl stress [19].

Allergic asthma and allergic rhinitis are generally associated with a predominant diffusion of Th2-lymphocytes, as well as the acute phase of atopic dermatitis. On the other hand, Th17 and Th1 lymphocytes are more expressed in other asthma phenotypes and to an advanced stage of atopic dermatitis. The role of oxidative stress is provided by the detection of increased oxidative stress end products in atopic patients. These products, like malondialdehyde (MDA), can affect the polarization of lymphocytes to Th2 and Th17 subsets [20,21], promoting the inflammation process. With an activity similar to that carried out by Th2 cells in the production of type 2 cytokines, like IL-5 and IL-13, there are group 2 innate lymphoid cells (ILC2) [22]. As the major ILC subset in the lung [23], they are continuously exposed to triggers and are therefore activated by a variety of factors, including IL-33, TSLP, and IL-25-induced endogenous reactive oxygen species [24].

Although hymenoptera venom allergy is not included among chronic inflammatory diseases, higher levels of advanced oxidation protein products were found in these patients than in the healthy controls, and this difference did not change during immunotherapy [25]. It is known that eosinophils are the most dominant inflammatory cells in allergic disease and, once activated, present an even greater ability of free oxygen radical synthesis than high concentrations of neutrophils [26].

### 1.3. Sex Differences, Oxidative Stress, and Allergy

**Figure 1:**
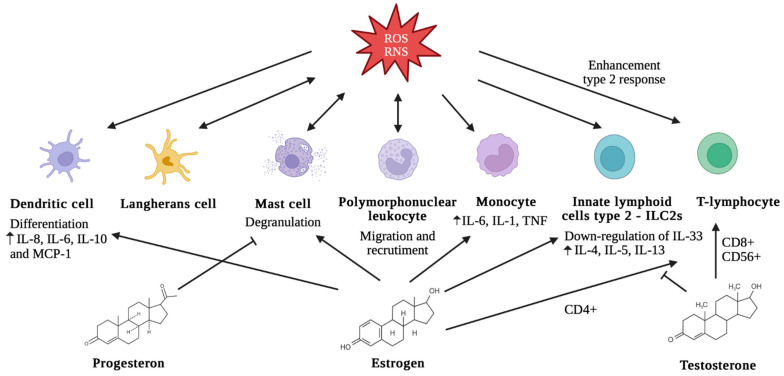
Interactions between oxidative stress, sex hormones, and immune regulation in allergy. ROS promote cytokine release, mast cell activation, and Th1/Th2 imbalance. Estrogens enhance immune and allergic responses, whereas androgens and progesterone exert immunosuppressive effects.

The action of sex hormones on the immune system (Figure 1) has been studied over the years: estrogens support a humoral response, autoimmunity [27,28], and are also involved in the activity of mast cells and type IV allergic reactions [29]. On the other hand, androgens, as well as progesterone and glucocorticoids, have an immunosuppressive effect [28], and act as a negative regulator of mast cell degranulation [30,31]. While monocyte and lymphocyte counts did not present particular differences between men and women, sex variations have been noted in lymphocyte subsets and natural killer cells. Men seem to present a higher percentage of natural killer cells and T-cytotoxic lymphocytes [32], and women have a greater proportion of T-helper cells [33]. It has been reported that Th1 responses are induced under low estrogen conditions and Th2 responses are induced under high estrogen conditions [34].

The cytokines level is also different between the two sexes based on the presence or not of a stimulus: in basal inflammation, men seem to present higher levels of them. This relationship is reversed in response to stimulus.

These differences are not only highlighted by the gap between males and females but there are also changes in the female menstrual cycle with different T-cell responses. This explains the variability of allergic symptoms in relation to the menstrual cycle, pregnancy, administration of oral anti-contraceptive drugs, and hormone replacement therapy [35].

According to the literature, women are found to have reduced markers of oxidative stress, explained by the greater generation of oxidative stress in men [13]. However, this hypothesis seems to depend on the cell type or tissue evaluated. The upregulation of glutathione metabolism and the lower NADPH oxidase activity by estrogen have been studied as possible factors in the greater resistance to diseases implicated with oxidative stress. Moreover, females have lower levels of p47, necessary for the assembly of the NADPH oxidase enzyme and of superoxide production independently of estrogen [36].

It is already known that estrogens exert antioxidant effects. In the postmenopausal period, women lose this protective effect and acquire a greater predisposition to develop anti-inflammatory and oxidative stress-related pathologies [37]. Surgical menopause is also associated with elevated production of oxidants as observed during ovary retention [38]. Estrogen replacement therapy (ERT) restores total plasma antioxidant capacity and decreases lipid peroxides [39]. Moreover, estrogen action has been linked to a greater synthesis of proinflammatory cytokines and mediators, like IL-6, IL-1, and tumor necrosis factor (TNF), in response to the stimulation of human monocytes. Consequently, removing endogenous estrogen reduces the proinflammatory response of the immunity system.

Estrogen receptors (ERa and ERb), activating the cAMP/protein kinase (PKA) signaling cascade and consequently the cAMP response element-binding protein (CREB) transcription factor, induce the expression of several antioxidants’ proteins (mitochondrial superoxide dismutase–MnSOD, heme-oxygenase-1–HO-1). Their role is also explained by genes encoding the antioxidant enzymes manganese-dependent superoxide dismutase (MnSOD) and catalase (CAT). Both ERs are present within the mitochondria, which reduce ROS production in response to estrogen, binding to mitochondrial DNA [40]. Indeed, ERb upregulates the expression of the SIRT3 protein, which translocates to the mitochondria and activates the MnSOD enzyme, increasing the detoxification of superoxide radicals. Two different studies reported higher SOD activity in female erythrocytes and plasma than male ones. This has been confirmed in many other species, including rats, that have higher SOD activity levels than males at the cardiac, pulmonary, and cerebral levels. Moreover, other experimental data have demonstrated that castration significantly reduced the levels of SOD activity in rats of both sex, indicating that sex hormones may be involved [41]. Mitochondrial biology is fundamental in maintaining redox homeostasis and significantly contributes to sex-dimorphic immune responses. Mitochondria, as the principal site of ROS generation during oxidative phosphorylation, exhibit activity that is finely regulated by sex hormones [42]. Estrogens promote mitochondrial biogenesis and bolster antioxidant defenses via activation of peroxisome proliferator-activated receptor gamma coactivator 1-alpha (PGC-1α) and upregulation of key antioxidant enzymes such as SOD and glutathione peroxidase (GPx) [43]. This regulation leads to a more balanced cellular redox state in females. Conversely, androgens are associated with enhanced oxidative metabolism and elevated ROS production [44]. Mitochondrial metabolism represents a pivotal nexus linking hormonal regulation with oxidative stress and immune function [45].

Glutathione plays a key role in cellular detoxification reactions and the regulation of the thiol-disulfide cellular status [46]. This tripeptide can exist intracellularly in an oxidized (GSSG) or reduced (GSH) state. Regarding GPX, estrogen, progesterone, and testosterone seem to control its activity. Indeed, a positive correlation between GPX and serum estrogen levels in both premenopausal and postmenopausal women with an ERT has been found. Total hysterectomy in premenopausal females reduced the expression of SOD and GPX but did not affect the expression of catalase [47]. Different studies have shown higher activity of GPX in female rats at the renal, cerebral, and hepatic level, while in male mice it is more present at the cardiac level [48]. In young girls, its activity was found to be higher in their blood compared to men [49]. The same study also highlighted a greater blood GSH/oxidized glutathione (GSSG) ratio in female adolescents [50]. Interestingly, hysterectomized women showed a decrease in GSH concentration and an increase in its oxidized form, subsequently normalized with an ERT. A recent report observed that surgical menopause and ERT did not alter CAT expression, which is important in the case of limited glutathione content or reduced GPX activity and plays a significant role in the development of tolerance to oxidative stress [51].

## 2. Sex Differences in Oxidative Stress and Allergic Disease

### 2.1. Allergic Rhinitis, Oxidative Stress, and Sex Differences

**Figure 2:**
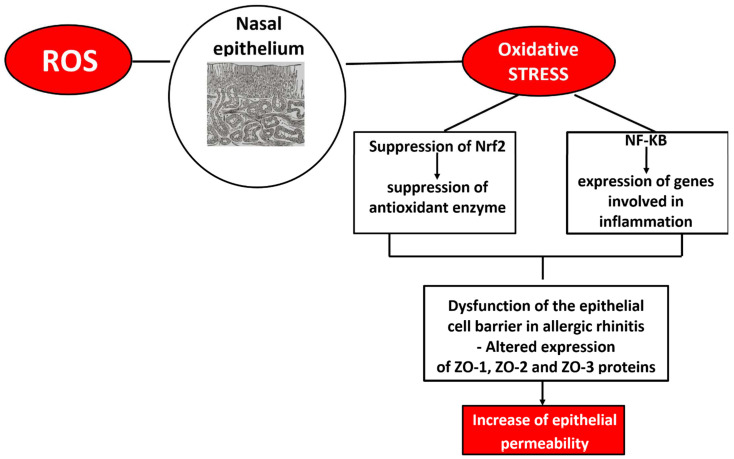
The main interactions between oxidative stress and nasal epithelium, with a consequent increase in epithelial permeability.

Allergic rhinitis (AR), an immunoglobulin E (IgE)-mediated inflammation of the nasal mucosa induced by inhaled allergens to which a patient is sensitized, is characterized by various symptoms, including repeated sneezing, nose itching, nasal obstruction, rhinorrhea, and ocular symptoms, involving inflammation of nasal mucosa [52] (Figure 2).

The development of AR involves complex mechanisms such as the interaction between genetic predisposition and environmental factors [53]. As reported in the literature and as stated above, there is a different prevalence of diseases related to atopic diseases in both sexes, and they change over the cycle of life. The differences between females and males are related to genes, hormones, immunology, and environment. Therefore, knowledge about the role of sex may be relevant in research and clinic, regarding AR [54]. Indeed, in the literature, it is reported that the prevalence of AR is higher in females than in males in adults; instead, childhood AR is more prevalent in males than in females with a change in adolescence [55]. The production of IgE antibodies by B cells through antigen presentation cell (APC) by dendritic cells, differentiation of CD4+ Th2 cell, and class switching in B cells are different between males and females, with a significantly higher prevalence of the disease in males in early age. After puberty, females have a higher expression of many allergic symptoms. Also, for AR the high expression of sex hormones during menarche, menses, pregnancy, or use of hormonal contraceptives may play a key role in the innate and adaptive immune system on nasal mucosal surfaces and IgE sensitization and allergy [56].

In allergic rhinitis, Nrf2/Keap1 (Kelch-like ECH-associated protein 1) and NF-B pathways are among the known signaling pathways in oxidative stress. In particular, these pathways play a key role as “molecular redox switches” in controlling activation or deactivation cycles and modulating system activities in a broad range of biological conditions [10]. In unstressed conditions, there is a suppression of Nrf2 in the transcriptional function due to ubiquitination and degradation by Keap1 [57]. Under oxidative stress, Keap1 is modified and no longer capable of ubiquitinating Nrf2, inducing the release of Nrf2 and its accumulation in the nucleus, which acts as a transcription factor to induce antioxidant and detoxication enzymes [58]. In this pathway, thioredoxin reductase 1 (TrxR1) plays a key role as a regulator for Nrf2 [59]. The role of thioredoxin-interacting protein (TXNIP) is evaluated in oxidative stress in allergic rhinitis [60].

Zhang W et al. reported significant alternations of sneezing, nasal rubbing, inflammatory cytokines, eosinophil numbers, thioredoxin-interacting protein (TXNIP), malondialdehyde (MDA), and superoxide dismutase (SOD) levels in resveratrol or N-acetylcysteine (NAC)-treated mice compared with untreated AR mice [60].

Moreover, many studies focused on the role of the NF-B pathway in the AR murine model [61]. In particular, it was reported that in the OVA-induced AR model, oxidative stress markers such as MDA level and Nrf2 and NF-B pathways are upregulated, with an association with clinical inflammatory signs, histopathological signs, and an increase in Th2 cytokine levels. These markers were downregulated after treatment with an antioxidant, mangiferin [60].

Moreover, oxidative stress induces dysfunction of the epithelial cell barrier in allergic rhinitis, with an altered expression of occludin and zonula occlude (ZO-1, ZO-2, and ZO-3) proteins and a consequent increase in epithelial permeability. Due to this, there is facilitation of absorption of allergens and harmful exogenous particles through the nasal epithelium [62].

In another study, the authors used the OVA-induced AR mouse model to evaluate epithelial cell permeability after administration of a possible antioxidant, Piper nigrum extract [63]. By enhancing the Nrf2 transcription factor pathway, an increase in anti-inflammation enzyme heme oxygenase (HO)-1 synthesis, inhibition of the degradation of ZO-1 and occludin, and an enhancement of the epithelial barrier integrity were all reported. Moreover, in mice treated with Piper nigrum extract the authors reported a reduction in the release of histamine from mast cells, nasal symptoms, and eosinophil infiltration in nasal lavage fluid and nasal tissue. Therefore, these results highlighted how the antioxidant treatment induces the cytoprotective function of the Nrf2 and HO-1 signaling pathways, inhibiting the disruption of tight junction proteins in the allergic rhinitis model [63].

### 2.2. Asthma, Oxidative Stress, and Sex Differences

Asthma is a common chronic inflammatory disease of the airways in which there is a complex interaction between susceptibility genes and environmental factors [64].

**Figure 3:**
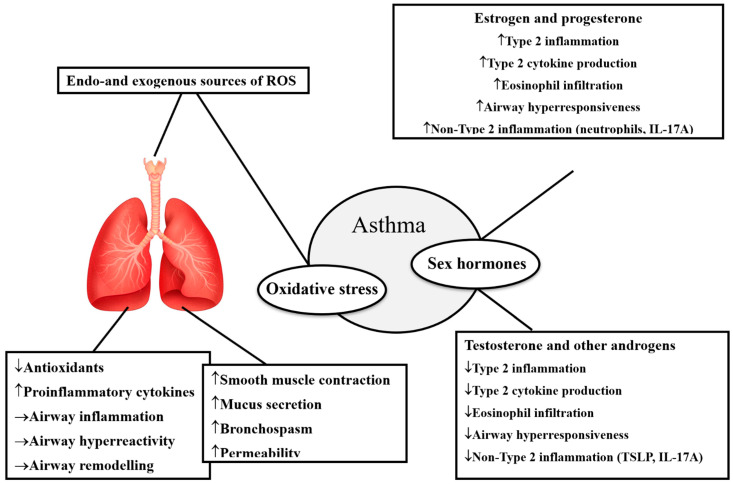
Schematic representation of the interplay between oxidative stress and sex hormones in asthma pathogenesis.

Oxidative stress plays a key role in the molecular mechanisms underlying asthma [65] (Figure 3). In the literature, it is reported that genes of antioxidant defense enzymes (ADE), such as glutamate cysteine ligase (GCLM), glutathione peroxidase (GPX1), catalase (CAT), myeloperoxidase (MPO), NADPH oxidase (CYBA, p22phox subunit), NAD(P)H: quinone oxidoreductase type 1 (NQO1), and microsomal epoxide hydrolase (EPHX1), are important determinants of genetic susceptibility to asthma in Russians [66]. ROS in exhaled air are very high, with a direct correlation with asthma severity. On the other hand, in the lungs of asthma patients, lower levels of antioxidant enzymes have been found (e.g., SOD, CAT and GPx). ROS are involved in the regulation of NF-κB and Nrf2 pathways, which are related to antioxidant, anti-inflammatory, and cytoprotective activities [67]. In the presence of oxidative stress, the Nrf2 signaling pathway is inhibited, with the consequent activation of NF-κB factor and mitogen-activated protein kinase (MAPK), triggering a proinflammatory response. An excess of oxidative stress inhibits the Th1 response and promotes the Th2 response, damaging proteins, lipids, and nucleic acid and inducing increased airway permeability, hyperresponsiveness, and mucus production [68]. Sex hormones tightly interact with redox-sensitive transcriptional regulators, modulating antioxidant and metabolic responses. Estrogens can activate the NRF2–ARE pathway, enhancing the expression of antioxidant enzymes such as HO-1, NQO1 (NAD(P)H:quinone oxidoreductase 1), and GCLC (Glutamate–cysteine ligase catalytic subunit), thereby contributing to cellular protection against oxidative stress [69,70]. Conversely, androgens may downregulate NRF2 activity, favoring higher ROS levels. Estrogen receptor signaling also cross-talks with FOXO (Forkhead box O) transcription factors, influencing apoptosis and immune tolerance, while hypoxic and inflammatory conditions modulated by sex hormones can alter HIF-1α stabilization and downstream metabolic adaptation [71].

Asthma is a complex multifactorial disease that is characterized by genetic heterogeneity related to the existence of shared genes that induce common susceptibility to the disease [66]. In particular, three ADE genes, which can be considered common susceptibility genes to asthma, such as glutathione-disulfide reductase (GSR), EPHX1, and GPX1, highlighted a relevant interaction in both variants of asthma in men and women (except for the GPX1 gene in nonallergic asthma in women) [66]. Sex differences in gene expression, epigenetic modifications, and responses to various environmental factors, such as SARS-CoV-2 infections, are associated with differences in asthma incidence, prevalence, and symptoms [72]. As reported above, asthma prevalence and severity are different in males versus females through various ages. The disease is prevalent in boys in children, while there is a prevalence and severity of the disease in women in adult age. For women, fluctuations in sex hormone levels during puberty, the menstrual cycle, and pregnancy are related to the pathogenesis of asthma [9]. Estrogen and progesterone fluctuate during the menstrual cycle, with a peak in the late follicular and mid-luteal phases. Decreased FEV1 and forced vital capacity (FVC) induce an increase in AHR and asthma-related healthcare utilization during the luteal phase [73]. In the literature, many studies highlight the role of hormone-dependent cyclic variations in asthma control and healthcare utilization. In particular, it has been reported that 20–40% of women with asthma had asthma exacerbations during pre- and peri-menstrual periods with a decrease in peak expiratory flow rates [74]. Moreover, during the premenstrual phase, it has been reported that women had increased sputum eosinophils and FENO compared to after menses [75]. In the literature, it is reported that the use of hormonal contraceptives plays a key role in impacting asthma incidence, prevalence, and control. Indeed, on analysis of data from serial national Scottish Health Surveys from 3257 premenopausal Scottish women, it was reported that hormonal contraceptives induce a reduction in asthma incidence and of asthma-related healthcare utilization, driven by a significant decrease in lean women and a reduction in wheezing in asthma patients [76]. Other analyses confirmed this result, in particular, that hormonal contraceptives decrease asthma incidence and asthma symptoms [77].

Estrogen signaling on ER-α induces an increase in AHR, IL-33 production, type 2 cytokine production, and eosinophil infiltration in the airways. However, the action of estrogen signaling on ER-β induces a reduction in AHR and eosinophil infiltration [78]. Testosterone and other androgens signaling through the androgen receptor (AR) induce a reduction in ILC2 proliferation, eosinophil infiltration, IL-33 and thymic stromal lymphopoietin (TSLP) production, and type 2 cytokine production [79]. A recent study [80] reported that AR signaling increased Treg suppression and decreased IL-33 production from airway epithelial cells in mouse models of airway inflammation. Moreover, in the same study, the authors also reported that dihydrotestosterone decreased fungal extract-induced IL-33 secretion from human bronchial epithelial cells [80].

Testosterone plays a key role in suppressing conventional dendritic cell responses and in reducing airway inflammation induced by house dust mites and *Alternaria* alternate fungal extract in murine models via the negative regulation of ILC2s [81]. The androgen dehydroepiandrosterone (DHEA) works to increase lung function, thereby decreasing asthma symptoms [82]. Indeed, it has been reported that decreasing testosterone levels in men older than 45 years was related to increased asthma prevalence [83].

Further studies are needed to determine how changes in sex hormones or the use of exogenous hormonal therapies alter asthma pathogenesis and response to current therapies, in particular, severe asthma biological therapies, to evaluate the efficacy of these therapeutics based on sex at various ages.

### 2.3. Urticaria, Oxidative Stress, and Sex Differences

Oxidative stress is a crucial event in chronic spontaneous urticaria (CSU), whose etiology is still unknown. CSU in characterized by the appearance of wheals and angioedema that persist for at least 6 weeks, without a specific cause [84]. The finding of high IgE antibodies, the high rate of association with Hashimoto’s thyroiditis, and an increased incidence of IgG antibodies directed to the FceRIa in a subpopulation of patients have supported the autoimmune hypothesis [85]. The activity of antioxidant enzymes and the level of lipid peroxidation end-product were found to be notably increased in the lesioned skin of CSU patients, as compared with the skin of healthy subjects. Indeed, it has been demonstrated that mast cells and basophils are capable of generating superoxide anion, which may regulate the process of activation and the release of cellular mediators in both physiological and pathological conditions [86]. An interesting study has analyzed the activities of manganese SOD, copper–zinc SOD, GPX, and CAT as indices of enzymatic antioxidant capacity, as well as MDA level as a lipid peroxidation marker in plasma and erythrocytes from CSU female patients with positive or negative response to autologous serum skin test without reporting statistically significant differences [87]. The unbalanced oxidant/antioxidant homeostasis has been more frequently reported in patients showing physical urticaria. Indeed, compared with controls, a decrease in plasma vitamin E, CAT, and GPX activities, and an increase in SOD activity and plasma polyunsaturated fatty acids, were registered [88]. These alterations may lead to an increased percentage of peroxidable compounds in the skin that make the cell more susceptible to oxidative agents (UV), explaining the patients’ urticarial response to stimuli. The reduction in vitamin E levels is probably due to the consumption during the lipoperoxidation process in cell membranes. Its replacement decreases the erythema response to UV, and its effect is enhanced when combined with ascorbic acid [89]. Experimental studies on mice confirmed the effect of supplementation of vitamin E on the cell-mediated immunity and cytokine production from Th1 lymphocytes to the detriment of those released by Th2 lymphocytes [90]. Elevated SOD activity observed in some patients represents an adaptative response to ROS overproduction. Converting the first product of one-electron reduction in molecular oxygen to hydrogen peroxide, which is subsequently removed by CAT, GPX, and metallothionein, SOD is considered the first line of defense against the damage of ROS [91].

Chronic urticaria is approximately twice more frequent in women than in men with higher rates of high disease activity, angioedema, refractoriness to treatment, and longer disease course [92]; however, sex-specific analysis among children under adolescent age showed no sex-specific differences [93]. As previously mentioned, sex hormones can regulate the activity of mast cells. Estrogens were found to promote histamine release of either rat mast cells or sensitized human basophils [94]. Zaitsu et al. highlighted that the physiologic concentration of estradiol induces the synthesis and release of mediators from mast cells via a non-genomic estrogen receptor-alpha and calcium influx, while progesterone and testosterone have the opposite effect [95]. Contrary to natural estrogens, the synthetic environmental ones (beta-hexachlorocyclohexane, polychlorinated biphenyls, and phytoestrogens) exert different influences on estrogen activities by mimicking or blocking their effects [96].

Hormonal fluctuations during the menstrual cycle play a role in urticaria expression, too; indeed, symptoms are aggravated during the premenstrual period [97]. Different studies demonstrated that Treg counts increase during the follicular phase of the menstrual cycle and undergo a significant decrease during the luteal phase; therefore, these patients probably have an autoimmune chronic spontaneous urticaria (CSU) subtype in which IgG autoantibodies activate the degranulation of mast cells [98]. Perhaps the lack of fluctuation in endogenous sex hormones in the postmenopausal period could be the reason for the reduction in wheal episodes compared to the perimenopausal and menopausal periods. It was also seen that by restoring hormonal imbalances with correct hormone replacement therapy, many women had fewer episodes of urticaria [98]. There are limited and contradictory observations regarding the correct role of oral contraceptives or hormone replacement therapy in the development of chronic urticaria and angioedema. Ornek et al., with a cross-sectional questionnaire study, analyzed the progression of urticaria according to different hormonal stages. Contrary to the expectations, the disease course did not change in the majority of patients during puberty, pregnancy, lactation, or menopause (100%, 96%, 84%, and 95.6%, respectively) [99]. The first study addressing the impact of pregnancy on the CU was the PREG-CU UCARE study [100] with a 51% improvement in CU symptoms, with 28.9% worsening and 20% unchanged. The reason for the increase in CU in the first and third trimesters was explained by the predominance of an immune response of TH1-type and the release of proinflammatory signals [101].

In addition, Kasperska-Zajac et al. [102] observed lower serum dehydroepiandrosterone sulfate (DHEA-S) concentration in patients with chronic urticaria, regardless of whether the autologous serum was positive or not, probably due to psychological distress [103]. DHEA-S has both immunomodulatory and anti-inflammatory properties, and its deficiency is possibly a permanent feature of some autoimmune diseases. In fact, after being released from the adrenal glands, DHEA is converted into other hormones, such as estrogen and testosterone, carrying out different effects depending on the districts examined [104].

### 2.4. Atopic Dermatitis, Oxidative Stress, and Sex Differences

Atopic dermatitis (AD) is a chronic relapsing skin disease with an increasing prevalence, ranging from 15 to 20% of children and up to 5% of adults. The etiopathogenesis is complex and multifactorial with genetic factors [105] and immunological and environmental aspects. The type of immune polarization is different based on the AD activity phase and also depends on the ethnicity and age considered, probably due to different hormonal regulation. In the acute phase, Th2 polarization is more prevalent with the release of many proinflammatory cytokines, like TNF, IL-4, IL-9, and IL-22, followed by Th17 and Th22 subtypes, while in the chronic phase, Th1 response is generally observed [106].

Chronic skin inflammation is associated with the overproduction of ROS, such as superoxide and hydrogen peroxide, that exceeds the defense capacity of the antioxidant system. Most frequently investigated biomarkers of oxidative stress in AD are searched for in blood and urine samples; less frequently, skin biopsy or cutaneous components are analyzed [107]. The most important searched biomarkers are the following: urinary 8-hydroxydeoxyguanosine (8-OHdG), which is a marker of oxidative DNA damage and repair; MDA, nitric oxide (NO), and 4-hydroxy-2-nonenal (HNE) that are end products of lipid peroxidation; and advanced oxidation protein products (AOPPs) and AGEs that derive from protein oxidation [108]. Data collected from more than 30 studies highlighted that 8-OHdG, nitrite/nitrate, and selenium are frequently altered in the urine of children with AD, with a positive correlation with the severity of cutaneous manifestations [93]. On the other hand, higher levels of serum MDA and lower levels of vitamins A, C, and E have been found in the serum of adult patients with AD [109]. Furthermore, Ji et al. found that the antioxidant factor heme oxygenase 1 was able to mitigate the development of AD manifestations in both mice models and AD patients [110].

GSH was evaluated in two different clinical studies with contrasting results: lower or equal quantities compared with healthy controls [109,111].

Recently, the aryl hydrocarbon receptor with its nuclear translocator (AhR/NT) has been given special attention as it promotes oxidative balance in a ligand-dependent manner in keratinocyte homeostasis [112]. Clinical trials have demonstrated therapeutic benefits with topical tapinarof, that is, a high affinity AhR ligand with antioxidative activity [113]. A consolidated role is played, instead, by the altered resident flora that stimulates IL-4 and the production of IgE, which causes skin inflammation. The production of ROS and RNS increases, enhancing cytokine and chemokine secretion. Even if the role of RNS in the pathogenesis of skin allergies needs further research, Nakai et al. found that nitrate concentrations were significantly higher in the urine of AD patients and correlated with disease progression. Among the human biological redox defense systems, bilirubin presents antioxidant actions, whose metabolites are biopterin (UBP), which are rapidly excreted into the urine. Shibama et al. [114] showed a possible involvement of UBP in AD pathogenesis with higher urinary levels than healthy controls.

Niwa et al. [115] conducted a study on skin biopsies showing increased free radicals’ production, resistance to substrate peroxidation, and higher antioxidant defense activity in AD patients compared to controls.

The prevalence of AD in childhood is slightly superior in males than females, but after puberty, this difference is reversed, and the immune responses of patients are closely connected with a hormonal trend. In individuals aged over 65 years, there are more diagnoses among males [116].

Female hormones mostly enhance the activities of Th2/regulatory T-cells (Treg) but suppress Th1/Th17. Therefore, estrogen has a positive impact on skin barrier integrity, while progesterone has detrimental effects, similar to those exerted by testosterone. Androgens like testosterone or dihydrotestosterone (DHT) present immunosuppressive activities, suppressing Th1, Th2, and Th17 cells, but promoting Treg activity [117]. However, the activity exerted by female sexual hormones is much more intense than that by male hormones [118]. Dehydroepiandrosterone (DHEA) enhances Th1 responses but suppresses Th2, but women are more susceptible to its influence, having higher levels of steroid sulfatase converting dehydroepiandrosterone sulfate DHEA-S to active DHEA [119].

A correlation has been pointed out between bilateral oophorectomy and a reduction in skin hydration as well as the integrity of the stratum corneum, with effects restored in the case of estrogen treatment [120].

### 2.5. Allergic Contact Dermatitis, Oxidative Stress, and Sex Differences

Contact dermatitis with both allergic and irritant etiologies is the most diffuse eczematous disease. Both forms show an inflammatory pathway with increased levels of inducible nitric oxide synthase (iNOS) protein expression [121], but in the irritant contact dermatitis (ICD) there is no underlying immunological process, and the trigger is the direct damage of the keratinocytes after contact with an irritant substance.

On the other hand, allergic contact dermatitis (ACD) displays a delayed-type hypersensitivity, which affects only susceptible individuals previously sensitized by allergen exposure [122]. It is a dermatosis mainly associated with Th1-Th17 phenotypes that shows a clear prevalence in female subjects. This result is influenced by several factors, both endogenous (skin structure, genetics, history of AD) and exogenous (sociocultural behavior and environmental exposure to allergens, product usage). Like in the other allergic skin disorders mentioned above, recent studies suggested the important roles of oxidative stress in the formation of ROS, RNS, and free radicals also in contact dermatitis. The first studies [123] documented elevated levels of proinflammatory factors, like IL-1, IL-6, and tumor necrosis factor (TNF)-a and ROS in both the initial allergen sensitization and the delayed response process [124]. The increase in TNF-a and MCP-1 levels could be explained by the activities of T-cells, macrophages, and keratinocytes [125]. Furthermore, not only irritant agents but also contact allergens can directly induce the expression of TNF-a and MCP-1 [126]. Indeed Martin et al. [127] demonstrated the expression of MCP-1 by basal keratinocytes and isolated dermal cells, which clearly anticipated the dermal accumulation of mononuclear cells during elicitation of ACD. Therefore, the imbalance of oxidative stress could be considered the starter point of the inflammatory cascade, which leads to the activation of transcription factors and the synthesis of inflammatory cytokines. Patients with a positive patch test to 5% nickel sulfate manifested increased tissue iron, reduced antioxidants poll of the skin, and an imbalance of oxidized/reduced GSH ratio, which characterize oxidative stress in the skin [128]. This result was confirmed by the increased serum concentration of a biomarker such as nitrosylated proteins in nickel-allergic patients after an oral nickel challenge [129]. According to Brans et al. [130], MnSOD polymorphism is associated with a higher risk of ACD among middle-aged women, but it is not an independent susceptibility factor for contact sensitization to para-phenylenediamine (PPD).

Kaur et al. [131] evaluated systemic levels of selected inflammatory markers, oxidative stress indices, and inflammation-related adipokines as well as their associations in patients with acute/subacute ACD involving approximately 5% of the body surface. Among the results, an increase in the level of TNF-a and the total peroxide concentration (TPX) was highlighted; on the other hand, the reduction in IL-10 values and total antioxidant capacity (TAC) was also noted. The decrease in the Th2-type regulatory cytokines such as IL-10, as reported in different studies, might indicate the downregulation activity in ACD [132]. Also, adiponectin level, a cytokine produced only by adipose tissue, was found to be elevated in these patients probably for a compensatory action [133]. It has an anti-inflammatory effect with the mediators’ production, such as IL-10 and IL-1 receptor antagonists and inhibition of TNF-a and NF-κB on endothelial cells [134]. Contrariwise, another product of the adipose tissue, like leptin, showed proinflammatory activity promoting Th1 cell polarization and monocyte recruitment [135]. Neither adiponectin nor leptin pointed out a significant correlation with the inflammation biomarkers, which therefore would require further investigation.

Greater female demand for patch tests, almost three times higher than males, and greater attention to skin health hide some of the differences in skin structure and physiology that are related to sex hormones [136]. Needless to say, different exposure to cosmetics and personal care products (PCPs) used in daily life promotes diverse patterns of contact sensitization, which affect various areas of the body.

Females demonstrated notable susceptibility to cosmetic allergens and their constituents, preservatives, and fragrances, and exhibited dermatitis more frequently on their face and hands [137]. On the contrary, male body areas mainly involved are the trunk and the extremities to some allergens like rubbers, resin, and chromate, probably linked to industrial and agricultural occupational exposures. Also, job categories are different between the sexes, with a reported higher prevalence for female healthcare professionals and male machinery mechanics and builders [138].

Boonchai et al. [136] conducted a retrospective chart review of patients undergoing patch testing, showing a higher female positive test rate than males (71.8–65%) and similar contact sensitizers for both sexes with different prevalence. Females presented more positive reactions to the following: neomycin, used in various pharmaceutical formulations [139] (topical corticosteroids, ophthalmic, ontological, and nasal solutions); nickel, which was 2.62 times higher than that in men; and formaldehyde and methylchloroisothiazolinone/methylisothiazolinone (MCI/MI), used as preservatives in PCPs and fragrance mix. Male sensitizing agents include carba mix, potassium dichromate, n-isopropyl-n-phenyl-4-phenylendiamine, epoxy resin, and parabens. These findings align with those reported in previous studies [140,141].

**Figure 4:**
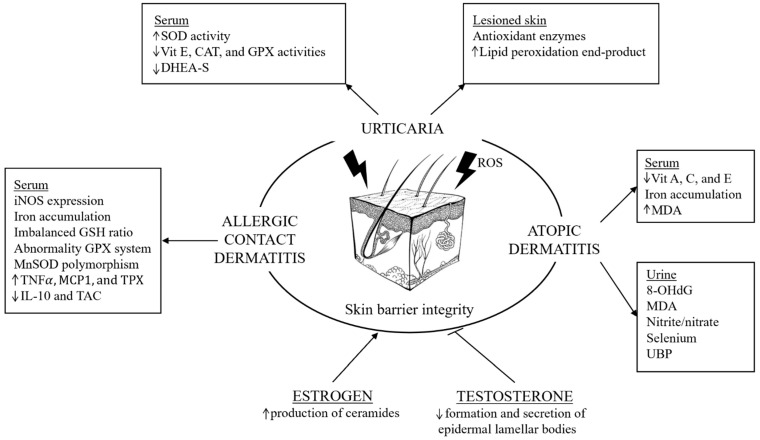
Sex hormones can regulate several skin processes relevant to the development of eczema, dermatitis, and urticarial manifestations.

Further studies are needed to understand sex differences in contact allergies to provide patients with personalized recommendations and promote the development of new and safe PCPs (Figure 4).

## 3. Sex Differences, Oxidative Stress, and Therapy

Many studies reported the potential role of dietary antioxidants as an alternative pharmacotherapy option for controlling these diseases analyzed above. Therefore, an emerging role in this field is played by nutraceuticals, which are substances contained in food with positive effects on health and on the treatment of various disorders [142]. The terms food supplements, dietetic, and functional foods are therefore possible synonyms and constitute the category of nutraceuticals, according to the European Nutraceutical Association (ENA) [143]. Vegetables and fruits have a wide range of different compounds, like alkaloids, polyphenols, and terpenoids, that are called phytochemicals, with positive effects on aging, inflammation, and chronic illnesses. The opportunity to combine conventional treatments with the use of natural products is now clear, improving the control of oxidative stress to reduce chronic inflammation [144].

In a 2017 study evaluating nasal epithelial cell barrier function after administration of sulforaphane [145], the authors reported that in house dust mite allergic rhinitis, the epithelial junction protein, zonula occludens (ZO)-1, was decreased but restored after Nrf2 activation by sulforaphane. Therefore, the authors reported how nasal epithelial cell barrier dysfunction in allergic rhinitis can be inhibited by activation of the Nrf2 pathway by treatment with sulforaphane [145]. In a double-blinded, randomized, placebo-controlled clinical trial, it was reported that sulforaphane induces a decrease in T2 cytokines such as IL-4, IL-5, and IL-13 in nasal cavity mucus of patients affected by allergic rhinitis. Moreover, after 3 weeks of sulforaphane treatment, total nasal symptom score (TNSS) and peak nasal inspiratory flow (PNIF) were improved [146].

In the literature, the effectiveness of resveratrol in reducing oxidative stress markers such as MDA is also highlighted [60]. In a double-blinded, randomized, placebo-controlled study, the authors reported that patients administered resveratrol had a decrease in nasal symptoms, serum IgE, IL-4, TNFa, and eosinophil levels compared to the placebo group [147]. Taurine was also studied in allergic rhinitis patients and in the OVA-induced murine allergic rhinitis model. In particular, taurine administration was related to a decrease in SOD3 level, nasal symptoms, inflammatory cytokine production, and inflammatory cell infiltration [148].

The latest research on the role of dietary supplementation on the progression of asthma severity showed conflicting results. The classic biomarkers of bronchial inflammation, such as FeNO, were not significantly correlated with a supplement of vitamins A and E, zinc, and copper [149]. However, Mabalirajan et al. [150] previously noticed that vitamin E attenuated the level of Th2 cytokines, suppressing airway hyperresponsiveness and mucus hypersecretion. Similar effects were found with resveratrol in experimental mouse models [151].

Neonatal studies pointed out that levels of selenium in maternal and cord blood have been associated with a lower incidence of wheezing [152]. The extracts, instead, of two plants such as Paeonia and Schisandra seemed to be able to decrease the levels of eosinophils, cytokines, and NF-κB [153].

Traditional Chinese medicine plays a relevant role in the modulation of multiple redox-sensitive signaling pathways. One study reported that zingerone, which belongs to the polyphenol group, induces a reduction in inflammation in asthma by acting on the AMPK/Nrf2/HO-1 signaling pathway [154]. In the literature, it is also reported that matrine, an alkaloid found in plants of the Sophora genus with antioxidant and anti-tumoral properties, can block asthma progression by downregulating IL-4, IL-13, STAT-6, and NF-κB [155]. Based on what is previously reported, it is well-known that the involvement of ILC2 in airway inflammation as well as IL-33-induced ROS is necessary for the activation of these cells. Therefore, it is easy to suppose how antioxidant therapy could improve this oxidative imbalance. It was seen, in murine models, that the activity of N-acetyl cysteine (NAC), a ROS scavenger, limits cytokine production, the proliferation of ILC2, and the associated eosinophilia [24].

Landi C et al. [156], after one month of benralizumab treatment, an interleukin-5 receptor alpha-directed cytolytic monoclonal antibody, detected upregulation of certain protein species of the antioxidant ceruloplasmin. In particular, the authors performed redox proteomics, which highlighted a lower oxidative burst after one month of benralizumab treatment than in the pre-treatment phase or after one month of mepolizumab therapy [156].

However, sex differences in biological asthma therapy have not been extensively studied. Retrospective analysis of anti-IgE treatment did not report differences between males and females in therapeutic response [157]. By now, regardless of the importance of other types of monoclonal therapies (i.e., anti-TSLP), a clear sex difference has not been assessed.

Sex differences in different asthma treatments may be due to compliance, variability in airway size and flow, and hormonal or sex-based pharmacogenetic and pharmacokinetic differences. Antileukotrienes showed better efficacy in women. This is probably due to the androgenic reduction in the 5-lipoxygenase activating protein (FLAP), which is required for the production of leukotrienes [158].

The involvement of oxidative stress in skin inflammation is almost clear in all the allergic skin disorders examined: urticaria, atopic dermatitis, and allergic contact dermatitis. Therefore, controlling oxidative stress in these skin diseases could be a supplementary therapeutic approach.

Considering desloratadine’s antioxidant activity in vitro, Cassano N. et al. [159] evaluated its effects on oxidative stress markers in patients with CIU, highlighting that desloratadine caused a relevant reduction in ROS levels and SOD activity (*p* < 0.005).

Even if the use of antioxidants in AD is controversial, often associated with an improvement in disease activity but not in pruritus, Yang et al. [160] conducted a systematic review and meta-analysis to evaluate the effectiveness and safety of antioxidant therapy in these patients. The results revealed that antioxidants could be a safe and efficient treatment, especially in children, in which a statistically significant difference was achieved (*p* = 0.02). Vitamin A, D, and E, combined with D, and topical vitamin B12 treatment significantly reduced the disease severity score (*p* < 0.05). More and more new topical antioxidant therapies are being developed: ROS/RNS production or release inhibitors or scavenging compounds [161,162]. A recent patent presented a topical formulation with a lipophilic cation mitochondrially targeted antioxidant compound that suppressed ROS formation by fibroblasts and ultraviolet radiation-induced activation of ERK in keratinocytes [163].

Natural compounds contained in the foods not only act on reducing exacerbations of skin disease with a steroid sparing but also have beneficial effects on comorbidities, such as psychological disorders [164]. Among natural products, some examples in the flavonoid group are represented by the extracts of wheatgrass [165] and Aronia melanocarpa [166], the topical application of Nymphoides peltate [167], the chlorogenic and caffeic acid present in Coffea arabica extract [168], the antioxidants found in blueberries and black rice [169], and quercetin [170]. All these substances improved the symptoms of AD by acting through different mechanisms: reducing the mRNA expression of iNOS, COX2, NO, and PGE2; increasing the expression of antioxidant protective factors (Nrf2 and HO-1); and promoting the expression of the barrier proteins (filaggrin, loricrin, and involucrin) [171].

Among the terpenes, another large class of natural compounds of animal and plant origin, astaxanthin is a carotenoid found in microalgae and crustacea whose liposomal formulation proved to be effective in the topical control of AD [172]. Knowledge of these factors, oxidative stress pathways, and sex differences could help clinical and therapeutic approaches to allergic disorders with personalized, precision management.

## 4. Conclusions

In this review, our analysis on the role of sex hormones and oxidative stress on the different expressions of allergic diseases allow us to highlight the differences in immune responses and regulation between the sexes.

Considering that atopic diseases such as allergic rhinitis, atopic dermatitis, and allergic asthma have a differential prevalence among women and men, with prevalence differences depending on age (affecting young males more than females, higher in post pubertal females where the incidence of allergies increases to become superior or equivalent to that reported in post pubertal males), we can understand how IgE levels and sensibilization are influenced by the menstrual cycle, and, in particular, by sex hormones. Immunological pathways driven by sex hormones induce the development of allergy, chronic inflammation, and autoimmune disorders, with a striking female sex bias as well-described above.

These considerations become essential for precise stratification of patients, identifying targeted treatments for a personalized medicine approach.

## Data Availability

Not applicable.
